# Complete Genome Sequence of the Cluster DJ Actinobacteriophage, Petito, isolated on the host *Gordonia rubripertinca*

**DOI:** 10.17912/micropub.biology.001456

**Published:** 2025-01-03

**Authors:** Elliot Benson, Micah Blount, Shria Chauhan, Jayden Ehrhart, Adelinn Foster, Abigail Ingber, Madeline Julian, Derika Kwansah, Trang Le, Emily May, Elizabeth Mazel, Esther Morency, Sierra Nelson, Casey O'Toole, Kaitlin Potter, Leandra Vita, Kirra Weigand, Denise Monti

**Affiliations:** 1 Hicks Honors College, University of North Florida, Jacksonville, Florida, United States

## Abstract

Gordonia phage Petito is a newly discovered siphovirus that infects *Gordonia rubripertincta* NRRL B-16540. The double-stranded DNA genome of this phage is 60,447 bp long with 93 predicted protein-coding genes and no tRNAs. Petito is a Cluster DJ phage.

**Figure 1. Transmission electron micrograph of Gordonia phage Petito.**
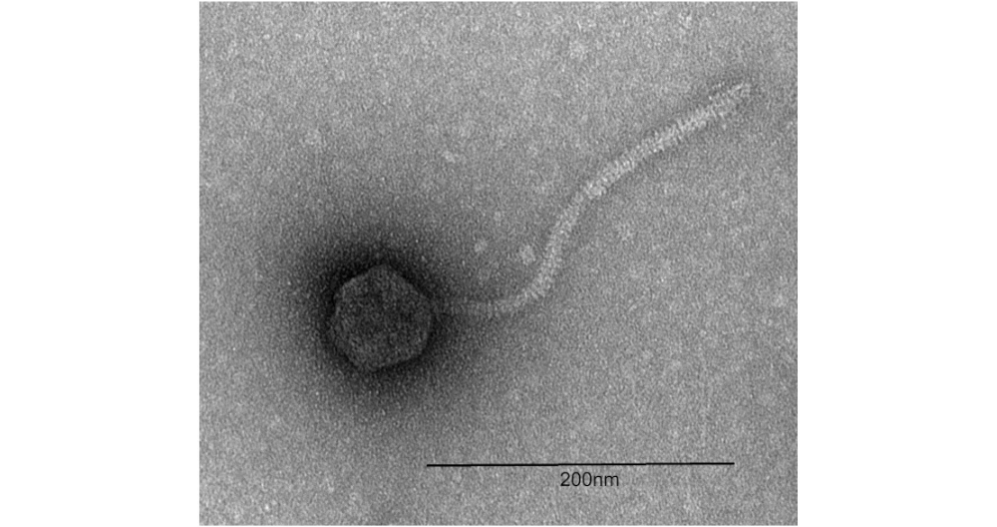
Phage lysate was negatively stained with 1% uranyl acetate and imaged with a Hitachi HT7800 120kV TEM and an AMT Nanospring15 B digital camera. Scale bar = 200nm.

## Description

Bacteria that comprise the genus *Gordonia* are metabolically diverse and have been implicated as possible agents for bioremediation (Arenskötter et al. 2004) . *Gordonia* spp. may have additional use in biotechnology as biomachines for synthesizing novel synthetic compounds. To better understand *Gordonia* spp. and to identify novel genes that may be exploited for synthetic biology, students in the Science Education Alliance-Phage Hunters Advancing Genomics and Evolutionary Science (SEA-PHAGES) program have isolated and characterized nearly 800 phages from nine species of *Gordonia* (phagesdb.org) (Russell 2016) . Here, we report the isolation and characterization of phage Petito isolated on the host, *Gordonia rubripertincta* .

Petito was isolated from soil collected from Ogier Gardens at the University of North Florida, Jacksonville, FL (30.27945 N, 81.51295 W). Soil samples were washed with Luria Broth (LB), incubated at room temperature with shaking for 1 h, and filtered through a 0.22µM filter. Soil extracts were enriched for phages infecting *G. rubripertincta* by inoculating the extracts with *G. rubripertincta* NRRL B-16540 and incubating for 48 hours at 30°C. Extracts were then filtered through a 0.22µM filter, 10-fold serially diluted, and plated with LB top agar containing *G. rubripertincta* NRRL B-16540. Phage replication formed small, clear plaques when incubated at 30°C for 24 hours. Petito was purified by three rounds of plaque purification assay (Zorawik 2024) , after which a viral lysate was prepared. Negatively stained transmission electron microscopy revealed siphoviral morphology with an icosahedral capsid (69 ± 2 nm diameter, n=2) and a noncontractile tail (252 ± 0.8 nm length, n=2).

Phage DNA was extracted using the Wizard DNA cleanup kit (Promega). A sequencing library was prepared using the NEB Ultra II Library Kit and sequenced using an Illumina MiSeq (v3 Reagents), yielding 298,983 single-end 150-based single-end reads (~719-fold coverage). Raw reads assembled and checked using Newbler v2.9 (Russell 2017) and Consed v29 (Gordon and Green 2013) with the default parameters yielded a single contig 60,447bp in length with 3' single-stranded overhangs (5' CGCCGTCCT).

DNAMaster (v5.23.6) and Phage Evidence Collection and Annotation Network (PECAAN) (https://blog.kbrinsgd.org/overview/) were used to annotate the genome. Ninety-three protein-coding genes were identified based on predictions by Glimmer v3.02 (Delcher et al. 2007) , GeneMark v2.0 (Besemer and Borodovsky 2005) , Starterator (v.575), and BLASTp against the Actinobacteriophage protein database. The genome was scanned for tRNAs using ARAGORN v1.2.38 (Laslett and Canback 2004) . No tRNAs were identified. Protein functions were assigned using HHPred (PDB_mmCIF70, SCOPe70, Pfam-A v36, NCBI Conserved Domain database) (Söding 2005), BLASTp v.2.14.1 alignments against the NCBI non-redundant protein sequences databases (https://blast.ncbi.nlm.nih.gov), and Phamerator (v575) (Cresawn et al., 2011) . Transmembrane domains were identified by DeepTMHMM v1.0.24 (https://dtu.biolibn.com/DeepTMHMM) (Hallgren et al. 2022) . Putative protein-coding genes were sorted into Phams using the pipeline PhaMMseqs (Gauthier, 2022) . All software was used with default parameters. Petito was assigned to Cluster DJ based on at least 35% gene content similarity (GCS) to sequenced phages in the Actinobacteriophage database (Gauthier and Hatfull, 2023) .

Similar to other phages assigned to Cluster DJ, all genes in Petito are transcribed in the forward direction. Twenty-four genes were assigned a putative function. Genes for structure, assembly, and lysis are encoded in the first third of the genome. Two copies of the major tail protein are encoded in the left arm of the genome along with other structure-related genes (e.g., minor tail protein, tail terminator). Of interest are two homologs of unknown function (Petito gp50 and gp61) encoded in the center of the genome that are also found in Arthrobacter, Microbacterium, and Streptomyces phages. A second pair of homologs of unknown function (Petito gp81 and gp82) are encoded in the last third of the genome. Unlike Petito gp50 and gp61, Petito gp81 and gp82 are specific to Cluster DJ phages.

**Nucleotide sequence accession numbers**

Petito is available at GenBank with Accession No. PP758912 and Sequence Read Archive (SRA) No. 30907824.
